# Speed and Oscillations: Medial Septum Integration of Attention and Navigation

**DOI:** 10.3389/fnsys.2017.00067

**Published:** 2017-09-20

**Authors:** Marian Tsanov

**Affiliations:** Trinity College Institute of Neuroscience, Trinity College Dublin Dublin, Ireland

**Keywords:** medial septum, theta rhythm, linear velocity, path integration, spatial learning

## Abstract

Several cortical and diencephalic limbic brain regions incorporate neurons that fire in correlation with the speed of whole-body motion, also known as linear velocity. Besides the field mapping and head-directional information, the linear velocity is among the major signals that guide animal’s spatial navigation. Large neuronal populations in the same limbic regions oscillate with theta rhythm during spatial navigation or attention episodes; and the frequency of theta also correlates with linear velocity. A functional similarity between these brain areas is that their inactivation impairs the ability to form new spatial memories; whereas an anatomical similarity is that they all receive projections from medial septum-diagonal band of Broca complex. We review recent findings supporting the model that septal theta rhythm integrates different sensorimotor signals necessary for spatial navigation. The medial septal is described here as a circuitry that mediates experience-dependent balance of sustained attention and path integration during navigation. We discuss the hypothesis that theta rhythm serves as a key mechanism for the aligning of intrinsic spatial representation to: (1) rapid change of position in the spatial environment; (2) continuous alteration of sensory signals throughout navigation; and (3) adapting levels of attentional behavior. The synchronization of these spatial, somatosensory and neuromodulatory signals is proposed here to be anatomically and physiologically mediated by the medial septum.

## Introduction

Anatomical dissociation of the brain structures mediating: (1) locomotion; (2) rhythmic theta oscillations; and (3) attentional control is not straightforward and easily distinctive. The reason for that is the evolution of these structures, which developed in a similar context—to guide navigation. The dysfunction of these brain areas impairs the motor, spatial or mnemonic components of navigation. The motor, oscillatory and attentional signals converge in several brain regions and one of the most potent convergence is evident in the medial septum. We will describe here each of the main systems that mediate locomotor activity, attentional control and theta rhythmic oscillations, and we will discuss how the convergence of these circuits in the septal region mediates path integration.

## Locomotor Circuits in The Brain

Locomotion in mammals is triggered by neural networks of spinal neurons located in the lumbar and cervical segments of the spinal cord. These networks are known as locomotor central pattern generators and they are sufficient to process continuous rhythmic muscle innervation towards the limbs, which constitutes the physiology of locomotion (Grillner and Zangger, 1979; Grillner et al., 1981; Kjaerulff and Kiehn, 1996; Marder and Calabrese, 1996; Whelan, 1996; Jordan, 1998). The primary mechanism of locomotor central pattern generation is based on rhythmic feedback loop involving excitatory and inhibitory feedback between the flexor and extensor spinal cord roots (Hinckley et al., 2005; Juvin et al., 2005; McCrea and Rybak, 2008; Frigon and Gossard, 2009). This basic evolutionary principle for whole-body movement of vertebrates demonstrates the fundamental link between locomotion and rhythmic neuronal activity.

**Figure 1:**
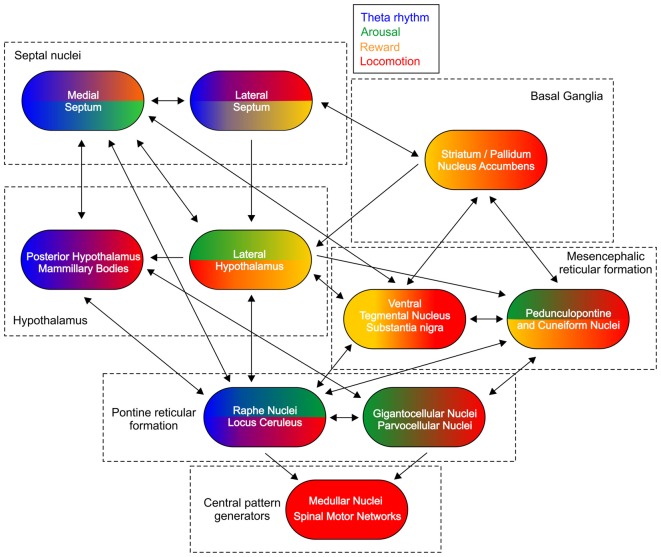
Subcortical control of locomotion. Schematic figure of the main connections between the structures involved in the subcortical control of locomotion. Color-coded representation depicts the functional integration of other major signals for each region. Theta rhythm is marked with blue color, arousal—green, reward—yellow and locomotion—red.

Hierarchically-organized brain networks provide descending projections to the central pattern generators for the initiation and the control of the locomotion (Figure 1). Despite the complexity of their connectivity and function the rhythmogenic structures share similar pattern of organization (Butera et al., 1999). On the top of this hierarchical network the medial septum displays a neuronal microarchitecture that leads to rhythmic pattern generation (Wang, 2002). The functional interactions between the supraspinal locomotor regions is complex as none of them serves as self-sufficient pace-maker of the locomotion, however all of them are involved in the regulation of whole-body motion. These brain areas are connected in a system where the signal processing reverberates across all levels of the network and propagates in both ascending and descending anatomical directions (Figure 1).

Electrical stimulation of midbrain nuclei is known to trigger locomotion in decerebrate cats (Shik et al., 1966). Subsequent line of research has led to the concept of mesencephalic locomotor region (MLR), which mediates the supraspinal generation of locomotor activity (Skinner and Garcia-Rill, 1984). The anatomical substrates of MLR are pedunculopontine nucleus, lateral cuneiform nucleus and midbrain extrapyramidal area (Sherman et al., 2015). The function of MLR as locomotor center has been considerably debated (Jordan, 1998) because selective activation of these nuclei in non-decerebrate mammals results in inconsistent locomotor outcome, affecting also the posture, gate, startle response, aversive behavior, cardiovascular and respiratory functions (DiMarco et al., 1983; Mitchell et al., 1988a,b; Mori et al., 1989; Depoortere et al., 1990; Kafkafi et al., 2003; Shafei and Nasimi, 2011; Gariépy et al., 2012; Sherman et al., 2015). The anatomical regions consisting MLR are also implicated in the behavioral state of arousal and rapid eye movement sleep (Brown et al., 2012; Ryczko and Dubuc, 2013; Van Dort et al., 2015; Goetz et al., 2016).

Activation of other brain regions is also linked to the initiation of locomotion. Electrical stimulation of the posterior hypothalamic nucleus induced wheel-running behavior and the running speed increased with increase in stimulation intensity (Bland and Vanderwolf, 1972; Bland et al., 2006). However, stimulation of hierarchically high-positioned brain structures does not lead to single output and the evaluation of the behavioral response after their stimulation is obscured by the functional complexity and diverse connectivity of these structures. The role of the regions comprising the supraspinal locomotor control is elucidated after their inactivation. While electrical stimulation of the medial septum fails to induce wheel-running behavior (Bland et al., 2006), the initiation of locomotion is blocked when the medial septum is pharmacologically inactivated (Oddie et al., 1996). The effect of septal complex on the locomotion is due to the extensive septal innervation of the pontine reticular formation (Swanson and Cowan, 1979; Kalén and Wiklund, 1989) and mesencephalic tegmental area (Satoh and Fibiger, 1986). Pharmacological inactivation or lesion of the mesencephalic reticular system (Shefchyk et al., 1984; Brudzynski et al., 1993; Sinnamon and Benaur, 1997) leads to suppression of locomotion. The experimental inactivation of the nigro-striatal pathways (Baron et al., 2002) widely supports the clinical manifestation of motor dysfunction, best described in the Parkinson’s disease (Albin et al., 1989). Damage to the lateral hypothalamus causes profound physical inactivity in rodents (Levitt and Teitelbaum, 1975; Hara et al., 2001). Major pathway from lateral septum to lateral hypothalamus regulates the speed of locomotion (Bender et al., 2015), and lesions of lateral septum lead to hyperactivity (Sheehan et al., 2004). Finally, the inactivation of medullar and pontine reticular formation, including receptor-specific glutamatergic, noradrenergic, dopaminergic and serotonergic antagonism abolishes the initiation and maintenance of whole-body motion (Jordan et al., 2008).

## Reinforcement Behavior

While electrical stimulation in medial septum has little effect on locomotor activity, the medial septum is widely recognized as a brain site for intracranial self-stimulation in standard operant conditioning situations. Self-stimulation behavior of animals with stimulation electrodes implanted in the medial parts of the septal complex revealed higher self-stimulation rates and lower self-stimulation thresholds compared to animals implanted in the lateral septum (Cazala et al., 1988). Electrical stimulation of septal region generates self-stimulation behavior, which does not interfere with body mobility and/or with the spatial orientation of the self-stimulated rodents (Olds and Milner, 1954). Therefore, septum-mediated behaviors, including locomotion and spatial navigation, are not generated only in the septal area but across wide network of cortical and subcortical structures. The reinforcement behavior during septal self-stimulation is likely to be mediated by the septo-hypothalamic pathway (Sinnamon, 1993). The lateral hypothalamus is a diencephalic brain area classically implicated in feeding and reward (DiLeone et al., 2003; Harris et al., 2005; Stuber and Wise, 2016). The lateral hypothalamus is densely connected to another brain region that is involved in reward-dependent behavior, namely the dopaminergic ventral tegmental area (Shizgal et al., 1980; Bielajew et al., 2000; Nieh et al., 2015). Implantation of stimulation electrodes in the lateral hypothalamus evokes even higher rates of self-stimulation compared to the septal implantations (Cazala et al., 1988). Ascending septal GABAergic fibers indicate hippocampal involvement in the self-stimulation paradigm. Transgenic J20 mice with diminished density of GABAergic terminals on hippocampal interneurons, displayed a delayed acquisition and a lower performance of septal self-stimulation compared to controls (Vega-Flores et al., 2014b). Furthermore, septal self-stimulation supresses the amplitude of Shaffer collaterals-triggered field excitatory postsynaptic potential in wild type mice, but not in J20 mice. The excitatory postsynaptic potentials induced at the CA3-CA1 synapse are proposed to reflect the learning process of brain stimulation reward paradigm (Vega-Flores et al., 2014a). These data show that the functional integrity of septo-hippocampal pathway is involved in the medial septum-evoked self-stimulation behavior. Early studies debated the contribution of the theta rhythm to the intracranial self-stimulation. No differences in rate of septal self-stimulation response have been observed between stimulation protocols that enhanced or attenuated hippocampal theta rhythm (Ball and Gray, 1971). However, later study found that septal self-stimulation phase-locked to theta rhythm resulted in higher number of lever presses (Buño and Velluti, 1977). The septo-hippocampal circuitry might be involved in the context-dependent learning during reinforcement tasks but not in the sensorimotor components of reward-seeking behavior. Another region may exert the attentional control on septal activity for the acquisition of motor activities in instrumental conditioning. While brief electrical stimulation of hippocampus has no effect on the acquisition of an operant conditioning task by observational learning, the same stimulation protocol applied to the medial prefrontal cortex prevents the proper motor sequence of pressing the lever and going to the feeder to collect the reward (a pellet of food) during the operant conditioning task (Jurado-Parras et al., 2012). The medial prefrontal cortex projects to the cholinergic neurons in medial septum (Gaykema et al., 1991) and the prefrontal innervation of septal area may represent an essential anatomical substrate of the attentional behavior. The spiking activity of population of neurons in the medial prefrontal cortex is shown to increase during attentional performance task (Gill et al., 2000). Prefrontal activation of cortical cholinergic inputs is then proposed to compose a top-down attention system, which augments sensorimotor information processing across multiple sensory and limbic areas via activation of cholinergic inputs (Sarter et al., 2001). Cholinergic neuromodulation exhibits a dual role in the locomotor and attentional systems in the brain, which we will discuss next.

## Attentional Control

Evolutionarily the acetylcholine has been established as the major neuromuscular signal transmitter. In vertebrates the motor neurons are cholinergic and they release acetylcholine at the neuromuscular junction to initiate a muscle contraction. The central nervous system evolved to control the initiation and duration of rhythmically-generated body movements from the spinal motor networks. With the evolution of the midbrain and forebrain the acetylcholine developed neuromodulatory role in sustaining of attention and promoting arousal (Himmelheber et al., 2000; Jones, 2005). The anatomical location of cholinergic centers of the brain is: (1) the basal forebrain including the medial septum/diagonal band of Broca complex and nucleus basalis (Semba and Fibiger, 1988; Semba, 2004); and (2) mesopontine tegmentum including pedunculopontine and laterodorsal tegmental nuclei (Mesulam et al., 1983; Lee et al., 1988). Cholinergic neurons are also distributed in the basal ganglia (Woolf and Butcher, 1981; Bolam et al., 1984) and the medial habenula (Cuello et al., 1978; Aizawa et al., 2012).

The nuclei in the mesopontine tegmentum contain a heterogeneous neuronal population including cholinergic, GABAergic and glutamatergic cells (Lee et al., 1988), which project in ascending and descending directions. The descending projections target the pontomedullary reticular formation and spinal motor networks (Rye et al., 1988; Semba et al., 1990). The ascending pathways target directly the septal complex (Woolf and Butcher, 1986; Hallanger et al., 1987; Hallanger and Wainer, 1988) or indirectly via the posterior and lateral hypothalamic nuclei (Woolf and Butcher, 1986; Oddie et al., 1994; Ford et al., 1995). The basal forebrain cholinergic region forms diverge cholinergic and GABAergic projections across several cortical areas. Medial septum innervates the limbic cortices (Amaral and Kurz, 1985; Wainer et al., 1985), while the nucleus basalis targets sensory, motor and association cortices (Woolf et al., 1983; Casamenti et al., 1986). This extensive efferent divergence of forebrain cholinergic center promotes its functional role to mediate attentional behavior (Sarter and Bruno, 2000).

The neurophysiological substrate of attention is a complex brain process involving several neuromodulators and multiple cortical and subcortical brain areas (Posner and Petersen, 1990). The cognitive structure of attention includes three modules: alertness (arousal networks), orientation (information from sensory input, multisensory integration and episodic memory formation), and executive control (decision making and motor precision) (Posner and Petersen, 1990; Fan et al., 2002). Behaviorally, attention allows the subject to obtain short- or long-term gains, by optimally sustaining the physiological needs of survival, food and reproduction, as well as novelty exploration and social interactions in mammals. This process involves the dopaminergic neuromodulatory systems mediating reward (Shohamy and Adcock, 2010). The septal cholinergic neurons are innervated by the dopaminergic tegmental inputs (Rutz et al., 2007; Watabe-Uchida et al., 2012; Zarrindast et al., 2012). Reciprocally, the cholinergic system regulates dopamine release from ventral tegmentum. The activation of acetylcholine projections from mesopontine tegmentum and basal forebrain influences the responsiveness of dopamine neurons, regulating the release of dopamine in the ventral tegmental area and substantia nigra (Mena-Segovia et al., 2004; Omelchenko and Sesack, 2006; Winn, 2006). This mutual interplay comprises a major subcortical component of the orienting attentional control (Mena-Segovia et al., 2008).

Large number of human clinical studies and animal psychopharmacological experiments on the cholinergic neuromodulation have implicated the acetylcholine as an essential neuromodulator of sustained attention (Sarter et al., 2001). Lesions of the basal forebrain potently downgrade the reaction times of rodents during spatial attention tasks (Robbins et al., 1989; Muir et al., 1994; Bushnell et al., 1998). Lesions of the cholinergic basal forebrain in monkeys disrupt attention during orientation task (Voytko et al., 1994). Reversible suppression of the neuronal activity in the basal forebrain of rats increased the number of errors in sustained attention performance tasks (Holley et al., 1995; Moore et al., 1995). Cortical efflux of acetylcholine is increased throughout the performance of sustained attention task (Himmelheber et al., 2000). Exposure to behaviorally relevant unconditioned or conditioned stimuli triggers attentional behavior and leads to augmentation of cortical acetylcholine levels (Inglis and Fibiger, 1995; Moore et al., 1995; Acquas et al., 1996; Himmelheber et al., 1998). Septal cholinergic neuromodulation mediates shifting states of the brain and attentional modulation particularly of the limbic system (Hasselmo, 2006; Lee and Dan, 2012), and facilitates hippocampus-dependent memory formation (Everitt and Robbins, 1997; Gold, 2004). The role of attention is essential for both encoding and retrieval of episodic memory (Fernandes et al., 2005). The variability of the hippocampal place cells’ spiking depends on the attentional behavioral state of the animals (Olypher et al., 2002) and the place fields are more directionally-selective when the rodents search for food presented in a particular goal location, compared to behavioral protocols where the food is dispersed throughout the recording arena (Markus et al., 1995).

The attentional effect of cholinergic neuromodulation is mediated through amplification of stimulus-related processing (McKenna et al., 1988; Tremblay et al., 1990; Murphy and Sillito, 1991) and increase of signal-to-noise ratios in the cortical target areas (Everitt and Robbins, 1997; Sarter and Bruno, 1997). Attention controls the variability of sensory signals and their integration in the limbic cortices through augmented neuronal synchronization during the perception of task-relevant stimuli. The neurophysiological substrate of attention involves increased power of neuronal oscillations and phase synchronization of neuronal activity (Muzzio et al., 2009). Synchronized population oscillatory increases the temporal synaptic interaction of large number of cells as the membrane depolarizations fluctuate synchronously for several pre- and postsynaptic neurons (Hasselmo et al., 2002; Axmacher et al., 2006). Such mechanism facilitates the neuronal processing, signal encoding, memory formation and consolidation (Singer, 1993; Salinas and Sejnowski, 2001). The frequencies of neuronal synchronization that mediate attentional signal processing range from slow (less than 1 Hz; Sirota and Buzsáki, 2005) to high gamma (up to 100 Hz) oscillations (Singer and Gray, 1995). The most potent rhythms expressed during attentive states of behavior are theta (5–12 Hz) and low gamma (30–60 Hz) oscillations (Buzsáki, 2010). Next, we will review the role of medial septum for the limbic oscillatory synchronization in the context of spatial navigation.

## Theta Oscillations and Linear Speed

For several decades the medial septum is identified as generator of theta rhythm (Petsche et al., 1962; Stumpf et al., 1962). Numerous findings established the central role of septal region as pace-maker of limbic theta oscillations, thoroughly examined in several fundamental reviews (Bland, 1986; Stewart and Fox, 1990; Vinogradova, 1995; Buzsáki, 2002). Despite the extensive research on septal rhythmogenesis it is still unclear whether theta only synchronizes limbic neuronal activity or if the theta cycle also carries particular information necessary for the hippocampal function. One of the correlates established for theta rhythm power is the linear velocity signal (Vanderwolf, 1969; Whishaw and Vanderwolf, 1973; McFarland et al., 1975). Information about the whole-body motion of the animals is one of the major candidates for theta signal processing (McFarland et al., 1975; Oddie and Bland, 1998; Wyble et al., 2004; Sinnamon, 2006; Geisler et al., 2007). The firing frequency of the cells in the medial septum is modulated by speed with 65% of the rhythmically-bursting septal cells show a significant correlation between the inter-burst frequency and the animal’s running speed (King et al., 1998). Neurons, with firing patterns that highly correlate to the running whole-body speed are detected in other brain regions; movement-related correlates of single-cell activity is observed in posterior hypothalamus, particularly in the medial mammillary nucleus (Sharp and Turner-Williams, 2005). A large portion of neurons in the interpeduncular and habenular nuclei also show a temporally-coarse correlation with running speed (Sharp et al., 2006). These nuclei are anatomically connected to the medial septum (Herkenham and Nauta, 1977; Groenewegen and Wouterlood, 1988) and functionally they are linked to the basal ganglia and the limbic system (Hikosaka et al., 2008).

Recent optogenetic studies shed more light on the integration of speed and theta from the septal region. Photostimulation of septal glutamatergic VGluT2 neurons triggered locomotion, with velocity and duration that were predicted by the spiking frequency and number of activated VGluT2 cells (Fuhrmann et al., 2015). The three main neuronal types in the septal complex are GABAergic (Freund and Antal, 1988), cholinergic (Amaral and Kurz, 1985) and glutamatergic (Colom et al., 2005). The bursting frequency of the GABAergic neurons is coupled to septo-hippocampal theta waves (Borhegyi et al., 2004; Bassant et al., 2005; Simon et al., 2006; Hangya et al., 2009), while the role of glutamatergic cells is recently linked to locomotion. The pre-motor activity of the septal VGluT2 neurons predicts the speed of the upcoming movement and also indicates the entrainment of hippocampal theta oscillations (Fuhrmann et al., 2015). These data show that the activity of VGLuT2 neurons mediates the switch from inactive behavior to locomotor activity and integrates whole-body motion with theta rhythm. The locomotion-correlated activity is not restricted to medial septum but also engages the hippocampal neurons. Locomotion-related firing of VGluT2 septo-hippocampal projections supresses the activity of alveus/oriens interneuron-mediated CA3-CA1 feedforward inhibition prior to the movement initiation. Therefore, the increased firing frequency of VGluT2 cells in the septal region augments the excitability of hippocampal pyramidal cells during navigation (Fuhrmann et al., 2015). Recent finding revealed the involvement of lateral septum in the integration of theta rhythm and locomotion. Optogenetic activation of lateral septum projections targeting lateral hypothalamus shows the role of descending septal pathway in theta-rhythmic regulation of locomotion (Bender et al., 2015). Lateral septal dysfunction is associated with hyperactivity (Sheehan et al., 2004) and a major projections from lateral septum target the lateral hypothalamus, which is part of the diencephalic locomotor circuitry (Sinnamon, 1993). Experiments using photostimulation-evoked regulation of hippocampal theta rhythm in combination with axonal chemogenetic or optogenetic inhibition of hippocampo-septal pathway demonstrate that lateral septum controls the variability and speed of running during exploratory behavior (Bender et al., 2015).

**Figure 2:**
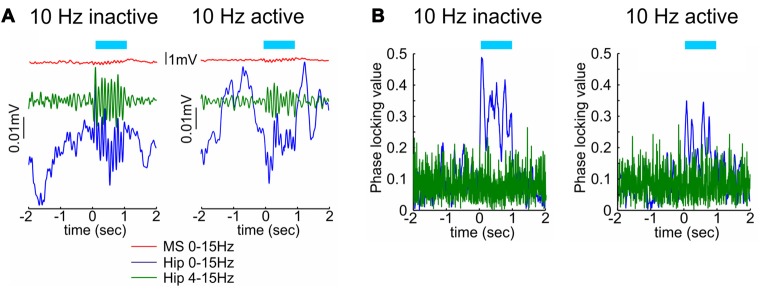
The effect of septal cholinergic activation on hippocampal oscillations depends on the behavioral state.** (A)** Optogenetic stimulation of cholinergic septal neurons after injection of cre-dependent virus in the medial septum of ChAT::Cre rats. Sample event related potentials (ERP) recorded in hippocampal CA1 region after 10 Hz septal ChAT photostimulation during inactive (left) and active (right) behavioral state. Upper red traces show ERP from medial septum, middle green traces represent ERP from hippocampus band-pass filtered (4–15 Hz) and lower blue traces represent the same hippocampal ERP after low-pass filtered (0–15 Hz). Time 0 indicates the delivery of the first train of 10 Hz stimulation protocol to medial septum. **(B)** Representative samples of phase-locking value for 10 Hz septal ChAT photostimulation during inactive (left) and active (right) behavioral state. Blue traces show the observed data, while the green values represent shuffled data (adapted from Mamad et al., 2015).

Optogenetic methodology allows us also to test the role of septal cholinergic neuromodulation of theta rhythm. Selective light-triggered depolarization of neurons expressing choline acetyltransferase (ChAT) evokes higher degree of spiking in the medial septum as well as in the dorsal hippocampus during inactive behavioral state, characterized with immobility, compared to active state, characterized with locomotor activity (Mamad et al., 2015). Optogenetic stimulation of septal ChAT neurons increases theta amplitude during the inactive states, while the same stimulating protocol during the active state results in significantly lower theta response (Figure 2A). Concurrent findings show that optogenetic stimulation of septal ChAT neurons promotes theta oscillations and suppresses sharp wave ripples and slow oscillations (Vandecasteele et al., 2014). This effect is expressed in experimental setup under anesthesia, whereas the same protocol elicits insignificant changes in any frequency range when the hippocampal oscillations are entrained by high theta power (Vandecasteele et al., 2014). Despite the behavioral dependency of cholinergic modulation on hippocampal oscillations, the optogenetic activation of septal ChAT neurons induces reliable reset of hippocampal theta rhythm and phase-locking synchronization of CA1 local filed potential (Figure 2B) in behaving rats (Mamad et al., 2015). These data suggest that the septal cholinergic innervation mediates the augmentation of hippocampal network activity during the transition from resting to waking state, but also cholinergic neuromodulation tunes the synchronization of limbic oscillations during active behavior. Earlier study in behaving animals showed that population of slow-spiking septal cells may promote the activation of limbic structures in all behavioral states (Zhang et al., 2011). Concurrently, septal activity precedes temporal power increase of hippocampal theta rhythm that continues for several seconds (Zhang et al., 2011). Inactivation of the septal cholinergic system decreases hippocampal theta power (Lee et al., 1994), however the impact of septal cholinergic activation on hippocampal theta oscillations is restricted to theta power but does not affect theta frequency (Vandecasteele et al., 2014; Mamad et al., 2015). Cholinergic neurons in the medial septum are considered to regulate the amplitude of theta, whereas septal GABAergic cells are believed to mediate the frequency of theta oscillations (Gerashchenko et al., 2001). Current models on theta-rhythm generation across the septo-hippocampal axis describe the interaction between septal and hippocampal GABAergic neurons as a key element in the generation and propagation of theta rhythm (Toth et al., 1997; Chapman and Lacaille, 1999; Buzsáki, 2002). The cholinergic neuromodulation mediates plasticity mechanisms during the theta episodes, supporting the formation of hippocampal memory traces (Huerta and Lisman, 1995; Hyman et al., 2003). Optogenetically-triggered septal cholinergic input, regulates the synaptic plasticity response of CA1 hippocampal neurons to the Schaffer collaterals activation, depending on the timing of cholinergic input relative to the efferent input (Gu and Yakel, 2011). These data confirm that septal cholinergic neurons are engaged in limbic neuronal synchronization and mediate synaptic plasticity during exploratory behavior. Overall, these optogenetic findings reveal that cholinergic projections support attentional states, while the glutamatergic projections from medial septum and GABAergic projections from lateral septum regulate locomotor behavior.

## Sensorimotor Signal Processing

**Figure 3:**
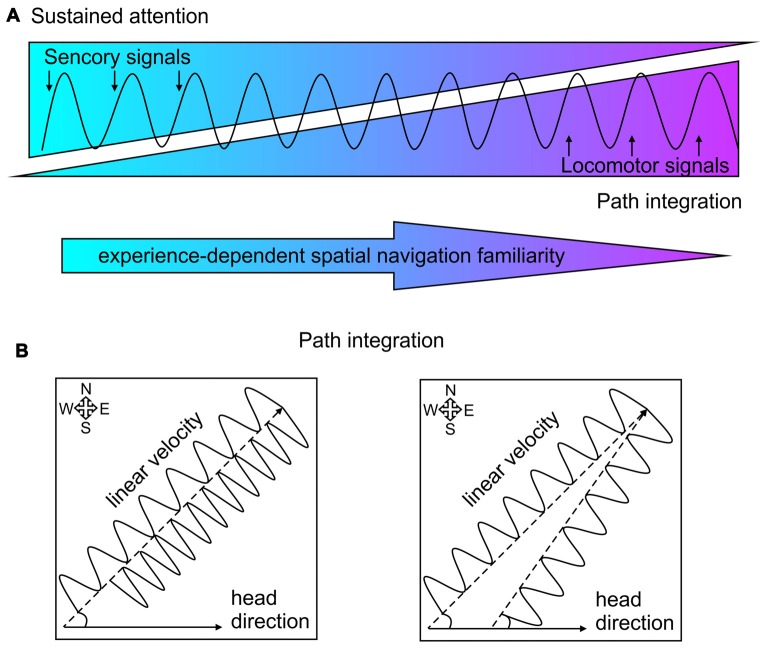
Theta rhythm incorporation of sensorimotor signals and path integration.** (A)** Schematic presentation of theta rhythm entrainment of sensorimotor signals. The balance between sensory signals (visualized in blue) and locomotor signals (purple) shifts over time after repeated navigation with increased spatial navigation familiarity (depicted with the arrow below). The sensory signals guide sustained attention, while locomotor signal mediate path integration. **(B)** Two-dimensional delineation of path integration. Left: spatial navigation towards a goal location with constant angular displacement (measured by the head direction) depends on the velocity value of the linear displacement. Lower linear velocity characterized with slower theta oscillation requires more time to reach the goal location (northeast corner), compared to higher linear velocity characterized with faster oscillation. Right: spatial navigation towards a goal location with constant linear displacement (characterized with the same oscillation frequency) depends on the directional value of the angular displacement from the starting position.

When we explore a novel environment (such as touristic trip to a new town) our navigation is guided by spatial cues and we focus our attention to remember them and use them for cue-based trajectory during a subsequent visit. When we are deeply familiar with particular trajectory (such as navigation to our workplace) our attention to the spatial cues is largely diminished and our cognition can focus on other tasks. In the first case our navigation relies of sustained attention, while in the second case our navigation relies on dead reckoning or path integration. Here, we discuss the hypothesis that septal complex is involved in both navigation strategies and septal theta rhythm integrates signals from both modalities (Figure 3A). When animals navigate in environment, the neuronal activity in the limbic formation rhythmically oscillates with a frequency of 5–12 Hz, an oscillatory range known as theta rhythm. Hippocampal theta amplitude and frequency depend on the linear velocity of locomotion and concurrently theta entrains the firing rate of the pyramidal neurons in hippocampus during navigation (Vanderwolf, 1969; Geisler et al., 2007). Hippocampal place cells, which comprise map-based spatial representation (O’Keefe, 1976), linearly enhance their spiking frequency with the locomotor speed (McNaughton et al., 1983; Wiener et al., 1989; Czurkó et al., 1999). The phase of spikes within each theta cycle depends on the spatial location of the animal, the path timing and the instantaneous firing rate of the neuron (O’Keefe and Recce, 1993; Harris et al., 2002; Mehta et al., 2002; Huxter et al., 2003). The firing rates of the inhibitory neurons in hippocampus are also regulated by locomotion-dependent suppression from the septal GABAergic cells (Fuhrmann et al., 2015).

Navigation is a process, which evolved to integrate sensorimotor signals: while the locomotion provides continuous spatial relocation, the sensory perception provides constant information about each location along the navigation trajectory. During spatial navigation the entorhinal and hippocampal regions integrate multimodal sensory information about the environmental stimuli (Knierim et al., 2013). Besides the visual and auditory signals, sensory information in the course of rodent locomotion is provided via rhythmic movements including sniffing and whisking (Welker et al., 1964). Limbic theta rhythm and whisking/sniffing are independent oscillators (Kepecs et al., 2006), but they synchronize during the sensory perception of task-relevant information (Berg et al., 2006; Buonviso et al., 2006). Therefore, the frequency of somatosensory and olfactory stimuli can be actively synchronized during behaviorally relevant spatial episodes. Medial septum synchronizes respiratory rate and theta oscillations and this integration allows the temporal alignment of intrinsic theta and extrinsic sensorimotor stimuli on each theta cycle. Single-unit recordings in behaving rats show that population of neurons in the medial septum can be phase-locked to the sniffing cycle (Tsanov et al., 2014). The firing of septal fast spiking and theta cells also express a temporal phase bias to the sniff cycle (Tsanov et al., 2014). The sniff cycle is considered as a coherent “timing unit” for olfactory signals processing across the limbic system (Buonviso et al., 2006). Both neuronal spiking and oscillations in olfactory bulb are phase-locked to respiratory cycle (Cang and Isaacson, 2003; Fantana et al., 2008), which can be synchronized with hippocampal theta oscillation (Macrides et al., 1982). Hippocampal theta rhythm and olfactory bulb oscillations correlate during olfactory discrimination tasks (Kay, 2005; Martin C. et al., 2007). These data demonstrate how septum-generated theta rhythm integrates global limbic oscillations and the activity of single sensory, motor and spatial neurons. Besides the integration of olfactory and somatosensory information, the septal complex controls the limbic incorporation of processed visual and auditory signals. Sustained attention performance is attributed to the sparse septal cholinergic innervation of multiple the primary, secondary and associated cortices where the cholinergic projections from the basal forebrain augment the identification and processing of sensory stimuli. Cholinergic neuromodulation increases the excitability of cortical cells to auditory stimuli, facilitating the detection and discrimination of auditory inputs (Weinberger, 1995). In visual cortex the cholinergic innervation enhances the intralaminar transfer of information between cortical columns including enhancement of higher perceptual processes (Xiang et al., 1998; Kimura et al., 1999). Acetylcholine evokes a composite modulation of the excitability of neuronal inputs and outputs to process behaviorally significant stimuli (Hasselmo and Bower, 1992; Tsanov, 2015). Septal cholinergic neuromodulation enhances the spiking of hippocampal pyramidal cells to excitatory afferent inputs (Krnjević et al., 1971; Cole and Nicoll, 1984), and concurrently suppresses the connectivity of glutamatergic synapses (Hounsgaard, 1978; Valentino and Dingledine, 1981; Dutar and Nicoll, 1988; Hasselmo and Bower, 1992). Overall, these findings demonstrate that during spatial navigation, the septal activity integrates sensorimotor signals and enhances the attentional perception of processed sensory information, and its long-term storage (Sarter et al., 2001).

## Theta Rhythm and Path Integration

When the information about particular trajectory is stored and reliably retrieved after repetitive exposure to the same path, the sustained attention towards spatial cues is gradually decreased (Figure 3A) with prevailing path integration (McNaughton et al., 2006; Taube, 2007). The process by which animals update their estimates of spatial position based on movement signals is known as path integration (Barlow, 1964; Etienne et al., 1996; Whishaw and Wallace, 2003). This process relies on continuous integration of movement information during the navigation (McNaughton et al., 1991; Etienne and Jeffery, 2004). The estimation one’s position of at any point can be calculated from (Figure 3B): (1) previously determined position (starting position); (2) linear displacement per time (whole-body speed); and (3) angular displacement (head direction; Whishaw and Gorny, 1999; Wallace et al., 2002; Stackman et al., 2003; Etienne et al., 2004; van der Meer et al., 2010; Valerio and Taube, 2012). Path integration relies on self-motion cues known as idiothetic signals. They include vestibular signals, which detect the head displacement; proprioception signals, which provide information from muscles and joints about limb position; and motor copy efference processed from the motor system during movements’ initiation and completion (Etienne et al., 1996; Knierim et al., 1996; McNaughton et al., 2006). The optic flow signals also sustain the continuity of spatial displacement, but this information is more relevant for animals (Collett and Collett, 2000) and humans (Wan et al., 2010), which exquisitely rely on visual cues for navigation. Nocturnal animals, however highly depend on the integration of self-movement cues during navigation. Rodents essentially depend on idiothetic signals to locate a present position (Whishaw and Gorny, 1999; Whishaw et al., 2001; Stackman et al., 2003).

Head-direction cells (Taube et al., 1990) process vestibular signals (Stackman and Taube, 1997; Golob and Taube, 1999; Bassett and Taube, 2001; Sharp et al., 2001b) and their role in the estimation of angular displacement is well-documented (Taube, 1998; Sharp et al., 2001a; Tsanov and O’Mara, 2015). The activity of these neurons, distributed across large network of diencephalic and cortical regions, is a key component of path integration (Muir and Taube, 2002; Taube, 2007). Linear displacement complements the estimation of relocation in the Cartesian plane and serves as major component of the path integration (Etienne and Jeffery, 2004; McNaughton et al., 2006). We know less about the brain systems that process linear velocity and the incorporation of the speed signal in the path integration. One of the major candidates that carry linear velocity signal is septum-generated theta rhythm (Figure 3B). The medial septum is key locus of speed processing due to the following findings: (1) septal neuronal activity correlates to speed (i.e., speed cells; King et al., 1998); (2) inactivation of medial septum abolishes locomotion (Oddie et al., 1996), while septal activation initiates locomotion (Fuhrmann et al., 2015); (3) lesions of medial septum disrupt path integration (Martin M. M. et al., 2007); (4) medial septum is pace-maker of limbic theta oscillations the amplitude and frequency of which depends on locomotor speed (Whishaw and Vanderwolf, 1973; McFarland et al., 1975); and (5) septum-generated theta oscillations depended on linear velocity (Geisler et al., 2007) and the length of traveled distance is directly related to the range of the theta cycle (Gupta et al., 2012). Locomotor speed is closely linked to changes in theta coherence along the limbic system (Hinman et al., 2011), but to become a reference for path integration the speed signal must be incorporated in the mapping of spatial representation. There are two neuronal substrates, which are proposed to integrate spatial signals and velocity-dependent theta oscillations: hippocampal place cells and entorhinal grid cells. Successful path integration depends on hippocampal (Whishaw et al., 1997, 2001) and entorhinal areas (McNaughton et al., 2006; Knierim et al., 2013), where the dysfunction of these limbic structures impairs motion-based navigation. The phase of the neuronal spikes of hippocampal place cells within the theta cycle correlates with the spatial location of the animal and the duration of the path (O’Keefe and Recce, 1993; Harris et al., 2002; Mehta et al., 2002). Theta entrainment of spiking activity of hippocampal interneurons and place cells is controlled by speed (Geisler et al., 2007). The speed-controlled assembly oscillator hypothesis proposes that the assemblies that receive similar information synchronize their activity with a common speed-dependent frequency (Buzsáki, 2010). An elegant research approach demonstrated the hippocampal neurons are capable of encoding time and distance as well as spatial location (Kraus et al., 2013). While rats were running in a treadmill they were trained to hold their position constant. Neurons recorded from dorsal hippocampus were strongly influenced by time and distance. The hippocampal cells encode not only time but also distance, with majority of neurons encoding both time and distance (Kraus et al., 2013). “Time cells” in hippocampus can be observed in experimental conditions when during locomotor activity the animal’s spatial position is consistently preserved (MacDonald et al., 2011). It is proposed that the medial entorhinal cortex provides spatial information to the hippocampal circuitry, while the lateral entorhinal inputs provide non-spatial input (Deshmukh and Knierim, 2011; Knierim et al., 2013; Van Cauter et al., 2013). Recordings from the medial entorhinal cortex demonstrated that grid cells and non-grid cells also encoded time and distance during animals’ running on a treadmill with different running speeds (Kraus et al., 2015). Majority of entorhinal grid cells and other neurons strongly signaled a both distance and time, with populations of cells influenced only by distance or time (Kraus et al., 2015). The neural activity in the entorhinal cortex, thus reflects computation of path distance based on the estimation of both time elapsed and running speed. Medial entorhinal cortex is proposed to mediate path integration computations using intrinsic, self-motion signals and extrinsic sensory information about geometry of the environment (Parron and Save, 2004; Knierim et al., 2013). The key role of the medial entorhinal cortex for the processing of linear velocity signal is revealed by the detection of speed cells (Sargolini et al., 2006; Kropff et al., 2015). Running speed is represented in the spiking frequency of a distinct population of entorhinal neurons different form other cell types such as grid, head-direction and border cells (Kropff et al., 2015). Grid cells, head-direction cells, border cells as well as speed cells within the entorhinal cortex collectively mediate the representation of the spatial position in the environment (Sargolini et al., 2006; Savelli et al., 2008; Solstad et al., 2008). All these neurons are strongly entrained by septum-generated theta oscillations (Giocomo et al., 2007; Brun et al., 2008; Giocomo and Hasselmo, 2008; Hafting et al., 2008). Theoretical models highlight the essential value of septal theta oscillations in spatial navigation where the activity of grid cells is dependent on theta rhythm entrainment and running speed (Hasselmo et al., 2007; Hasselmo, 2013; Shay et al., 2016). Passive transport abolishes velocity modulation of theta rhythmicity and disrupts the grid-like firing pattern of grid cells (Winter et al., 2015). The key role of medial septum for the integration of space and distance is demonstrated in experiments with inactivation of septal region. Pharmacologically-induced suppression of septal activity not only abolishes theta rhythm in the entorhinal–hippocampal network (Rawlins et al., 1979; Mitchell et al., 1982) but also impairs the formation of the grid cells in the entorhinal cortex (Brandon et al., 2011; Koenig et al., 2011). Behaviorally, medial septum inactivation results in impaired spatial learning (Mitchell et al., 1982; Mizumori et al., 1990; Lecourtier et al., 2011) and abolished path integration (Martin M. M. et al., 2007; Jacob et al., 2017). Excitotoxic lesions of medial septum or entorhinal cortex (but not dorsal hippocampus), attenuate the ability of rats to estimate linear distances based on self-movement information (Jacob et al., 2017). Medial septum inactivation impairs animals’ capability to run a specific distance in a linear track, using the information generated only by their own displacements (Jacob et al., 2017). Finally, septal lesions lead to deficits in self-motion based navigation and variability of temporal pacing of linear speeds (Martin M. M. et al., 2007).

## Conclusion

The contemporary understanding of medial septum is that this region does not simply generate rhythmic oscillations but also integrates variety of processed sensorimotor signals. We know now that sustained attention and spatial navigation crucially depend on the activity of functionally intact medial septum. The widespread cholinergic projections mediate increased neuronal responsiveness for the processing of sensory stimuli during transition from inactive to vigilant behavioral states. Concurrently, the septal inhibitory projections provide functional synchronization across several limbic regions facilitating synaptic transmission and plasticity. There is growing evidence for the significance of septal nuclei in the organization of spatial navigation. One of the main questions that we must address to properly comprehend the role of medial septum is how we can identify different neurophysiological components that combine together to form a distinct behavior. Simple navigation of a rodent in plain environment is complex behavior that involves whole-body motion, sensory perception, spatial orientation, value-dependent evaluation of the environment, awareness of environmental novelty. Despite the efforts of the researchers to separate each of these individual elements of navigation the brain has evolved to process them in parallel and to integrate their signals. Major candidate of this integration is the septal region where the neuronal activity responds to the perception of sensory stimuli, locomotion, arousal and reward. This polyvalent information is entrained in rhythmic theta oscillations, allowing for the theta cycle to serve as a common unit of composite signals. Another dimension that we have to consider when examine navigation is time. Repeated navigation is differently processed by the brain over time. With the increase of familiarity of particular environment the attentional perception of sensory stimuli is gradually replaced by the reliance on locomotor idiothetic signals. This review presented the hypothesis that repeated spatial navigation shifts the information content of theta cycle from sensory to motor signals propagation. We propose here that the septal region provides a dynamic balance between sustained attention and path integration during navigation. In summary, we can firmly outline the medial septum as a brain circuitry that rhythmically integrates processed sensory and motor signals necessary for navigation.

## Author Contributions

MT wrote the review.

## Conflict of Interest Statement

The author declares that the research was conducted in the absence of any commercial or financial relationships that could be construed as a potential conflict of interest.
